# Lentiviral-mediated panErbB CAR-T cell therapy against head and neck squamous cell carcinomas for patients with Fanconi anemia

**DOI:** 10.1016/j.omton.2025.201060

**Published:** 2025-09-22

**Authors:** Andrea López, David Charbonnier, Paula Vela, Begoña Díez, Paula Río, Rebeca Sánchez, Omaira Alberquilla, Beatriz Martín-Antonio, Jordi Minguillón, Esperanza Esquinas, Ramón García-Escudero, Ricardo Errazquin, Sonia Del Marro, Ania Pascual, Corina Lorz, Ángeles Juarranz, Andrea Barahona, Judith Balmaña, John Maher, Juan A. Bueren, José Antonio Casado

**Affiliations:** 1 Biomedical Innovation Unit, Centro de Investigaciones Energéticas, Medioambientales y Tecnológicas (CIEMAT), Madrid, Spain; 2 Centro de Investigación Biomédica en Red de Enfermedades Raras (CIBERER), Madrid, Spain; 3 Instituto de Investigaciones Sanitarias, Fundación Jiménez Díaz, Madrid, Spain; 4 Instituto de Salud Carlos III, Madrid, Spain; 5 IdiPAZ-CNIO Translational Research Unit in Pediatric Hemato-Oncology, La Paz University Hospital Research Institute, Spanish National Cancer Center, Madrid, Spain; 6 Instituto de Investigación Sanitaria Hospital 12 de Octubre (imas12), Madrid, Spain; 7 Centro de Investigación Biomédica en Red de Cáncer (CIBERONC), Madrid, Spain; 8 Departmento de Biología, Universidad Autónoma de Madrid, Madrid, Spain; 9 Departmento de Dermatología Experimental and Biología Cutánea, Instituto Ramón y Cajal de Investigación, IRYCIS, Madrid, Spain; 10 Vall d'Hebron Insititute of Oncology (VHIO) and Medical Oncology Department Hospital Vall d’Hebron, Barcelona, Spain; 11 King’s College London, School of Cancer and Pharmaceutical Sciences, CAR Mechanics Lab, Guy’s Cancer Centre, Great Maze Pond, London SE1 9RT, UK; 12 Leucid Bio Ltd., Guy’s Hospital, Great Maze Pond, London SE1 9RT, UK; 13 Department of Immunology, Eastbourne Hospital, Kings Drive, Eastbourne, East Sussex BN21 2UD, UK

**Keywords:** MT: Regular Issue, Fanconi anemia, head and neck squamous carcinoma, CAR-T cells, immunotherapy, ErbB receptors, EGFR

## Abstract

Fanconi anemia (FA) is a DNA repair syndrome characterized by bone marrow failure and cancer predisposition, including acute myeloid leukemia and solid tumors such as head and neck squamous cell carcinoma (HNSCC). Due to the exacerbated toxicity of radio-chemotherapy in FA patients with HNSCC, there is an urgent need of safer and more efficient antitumoral therapies for these patients, such as those based on chimeric antigen receptor (CAR)-T cells. Here, we show that HNSCC cell lines from both the general population and patients with FA express ErbB family members, which can be recognized by the T1E panErbB ligand. The generation of a lentiviral vector encoding for a second-generation T1E-CAR allowed us to generate panErbB CAR-T cells from healthy donors (HDs) and patients with FA. Despite the molecular and cellular defects characteristic of FA cells, a similar efficacy of CAR-T generation was observed, regardless of the donor origin. In all cases, panErbB CAR-T cells exerted potent cytotoxicity against all HNSCC cell lines tested *in vitro*. In addition, intratumoral administration of these CAR-T cells in HNSCC xenografts markedly reduced tumor growth. These preclinical results suggest that panErbB CAR-T cells would represent a safe, non-genotoxic therapy for HNSCC, with particular applicability for patients with FA.

## Graphical abstract

**Figure:**
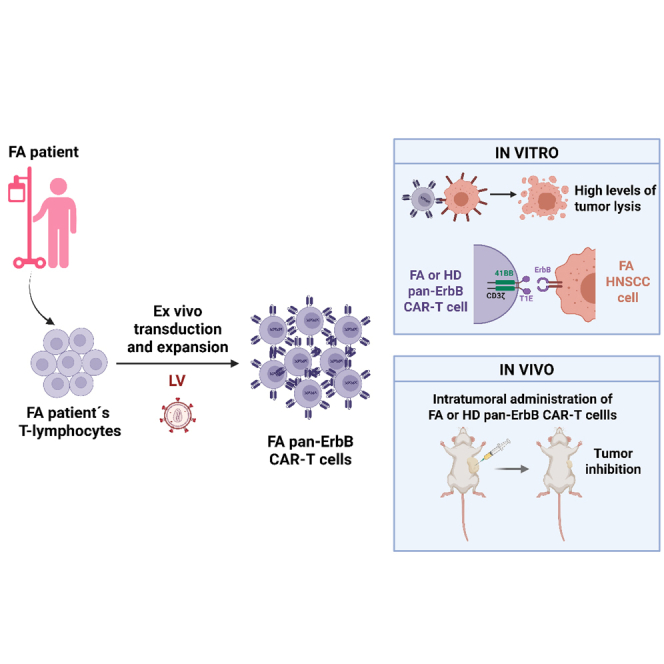

## Teaser

PanErbB CAR-T cells generated from both healthy donors and Fanconi anemia patients efficiently target HNSCC cell lines *in vitro* and suppress tumor growth in xenografts. These findings highlight the potential of non-genotoxic CAR-T therapies as safe and effective alternatives for FA-associated head and neck cancer.

## Introduction

Fanconi anemia (FA) is an inherited genetic disorder associated with mutations in any of the 23 FA/BRCA genes so far discovered.^1^ These proteins participate in the FA/BRCA pathway, which plays a crucial role in maintaining the genomic stability and the repair of DNA interstrand cross-links.^2^^,^^3^ Mutations in FA genes are also involved in several other molecular and cellular alterations, including oxidative stress,^4^ mitochondrial dysfunction,^5^ hypersensitivity to inflammatory cytokines,^6^ and up-regulation of NKG2D ligands.^7^ Clinically, FA is associated with congenital malformations, bone marrow failure (BMF), and a heightened predisposition to acute myeloid leukemia and solid tumors, mainly head and neck squamous cell carcinoma (HNSCC), whose incidence in patients with FA is approximately 500–1,000 times higher than in the general population.^8^^,^^9^^,^^10^

Currently, the only established curative treatment for BMF in patients with FA is the allogeneic hematopoietic stem cell (HSC) transplantation (HSCT) from a healthy donor, following conditioning treatments with chemotherapy and in some instances also radiotherapy.^11^ Several studies have reported that the incidence of HNSCC in transplanted FA patients is further increased with respect to non-transplanted patients, probably due to inflammatory processes associated with graft-versus-host disease and also the use of genotoxic conditioning agents.^8^^,^^12^^,^^13^ In addition to HSCT, we have very recently shown that the infusion of autologous gene-corrected HSCs in non-conditioned patients with FA facilitates the progressive engraftment of gene-corrected cells, as well as the correction of BMF.^14^^,^^15^ The absence of conditioning and the use of autologous HSCs strongly suggest that this new BMF therapy will contribute to prevent the increased incidence of HNSCCs reported in transplanted FA patients.^16^

HNSCC diagnosed in early stages (I and II) can be efficiently treated in the general population using surgery or radiotherapy, generally achieving high cure rates. Nonetheless, diagnosis commonly occurs at advanced stages (III and IV), which demand more aggressive therapies and result in a more limited outcome. Due to the very high incidence of HNSCCs in patients with FA, oral noninvasive screening is highly recommended to prevent the diagnosis of these tumors at advanced stages.^17^ In these cases, the high susceptibility of FA individuals to radiation and chemotherapy significantly restricts therapeutic options for these patients, leading to a reduced overall survival.^18^^,^^19^ These observations underscore the urgency to develop more efficient and safer therapeutic strategies for HNSCCs, very particularly in patients with FA. Innovative immunotherapies, such as chimeric antigen receptor T cell (CAR-T) therapies targeting HNSCC antigens, thus constitute non-genotoxic therapeutic alternatives to address this critical unmet need in patients with FA.

CAR-T cell immunotherapy has demonstrated very positive clinical responses in patients with specific hematological malignancies, particularly B cell leukemias and lymphomas,^20^ although its effectiveness against solid tumors remains a significant challenge.^21^ With the purpose of developing a CAR-T cell therapy against HNSCCs occurring in patients with FA, we aimed at targeting ErbB family members, whose expression in HNSCCs from the general population has already been shown.^22^^,^^23^^,^^24^ These members include ErbB1 (epidermal growth factor receptor [EGFR]), ErbB2 (HER2), ErbB3, and ErbB4, which form functional dimers that bind different ligands required for the survival and proliferation of different cell types, including HNSCCs. This was the basis of the use of anti-EGFR monoclonal antibodies, such as cetuximab, for the treatment of HNSCCs in the general population.^25^^,^^26^^,^^27^

To target HNSCCs, a CAR-based therapeutic approach defined as T4 immunotherapy was previously developed using a gamma-retroviral vector (RV) as the vehicle of the CAR construct. This vector mediates the expression of a promiscuous ErbB ligand (T1E), which recognizes 8 out of 10 possible ErbB homo- and heterodimers, only sparing ErbB2 and ErbB3 homodimers.^28^ A phase 1 clinical trial consisting of the intratumoral administration of T1E CAR-T cells was developed at Guy’s Hospital London in patients with locally advanced or recurrent HNSCC—none of whom had FA—and who were unsuited to any conventional form of therapy.^29^ Interim analyses of this clinical trial showed the safety of the intratumoral administration of panErbB CAR-T cells and even disease stabilization in some patients.^30^

Based on the preclinical and clinical studies conducted with T1E RV-mediated CAR-T cells, in this study, we generated a lentiviral vector (LV) carrying the T1E CAR sequence aiming at developing a novel panErbB CAR-T cell therapy as a non-genotoxic treatment for HNSCCs, which would have a particular impact in patients with FA. This would represent the first CAR-T cell therapy designed for FA patients with HNSCCs, addressing a critical gap in the therapeutic options available for this high-risk group of patients. Since this therapy should consider the treatment of both untransplanted and also transplanted patients with FA, we comparatively investigated differences in both the generation and the antitumoral properties of LV-based panErbB CAR-T cells derived from healthy donors (HDs) and also from these two groups of patients with FA.

Our preclinical studies suggest that designed panErbB CAR-T cells should constitute an effective non-genotoxic immunotherapy against FA HNSCCs, thus offering a potentially safer therapeutic alternative tailored to the unique needs of this vulnerable population.

## Results

### FA-proficient and deficient HNSCC cell lines consistently express high levels of ErbB1 and ErbB2 receptors

**Figure 1:**
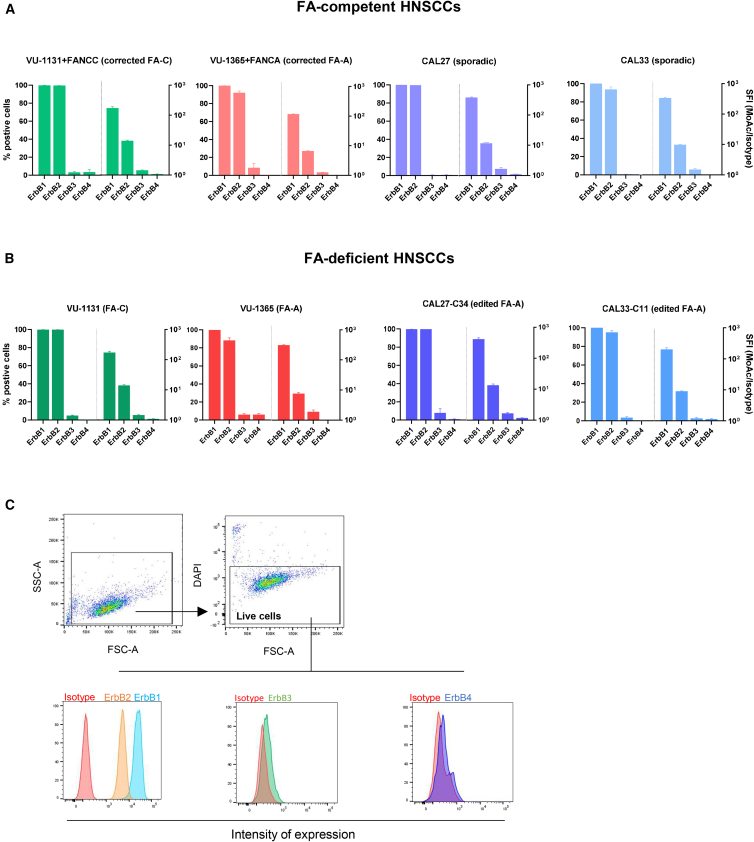
Analysis of the membrane expression of ErbB1, ErbB2, ErbB3, and ErbB4 molecules in FA-proficient and FA-deficient HNSCC cell lines Left and right axes represent, respectively, the percentage of ErbB positive cells and the specific fluorescence intensity (SFI) corresponding to each ErbB receptor. (A) FA-proficient HNSCCs cell lines. (B) FA-deficient HNSCCs cell lines. SFI expression levels of the different ErbB members were obtained as the ratio between the mean fluorescence intensity (MFI) after staining with the corresponding monoclonal antibody (MoAb) relative to MFI obtained with the control isotype. Bars show the mean ± SEM (*n* = 3). (C) Representative flow cytometry analysis of ErbB1-ErbB4 receptors in HNSCCs (VU-1131). FA-A and FA-C represent FA complementation groups with mutations in *FANCA* and *FANCC,* respectively.

To investigate the expression of ErbB molecules (ErbB1–ErbB4) in the membrane of HNSCCs proficient in the FA pathway (FA-proficient), cell lines from patients with sporadic cancer (CAL27 and CAL33), as well as gene-corrected HNSCCs cell lines from patients with FA (see materials and methods section; VU-1131+FANCC and VU-1365+FANCA) were used. In these studies, FA-deficient HNSCCs were also investigated. In this case, HNSCC cell lines generated from patients with FA (VU-1131 and VU-1365) and also non-FA HNSCCs genetically engineered to inactivate *FANCA* were used (see materials and methods section; CAL27-C34 and CAL33-C11). Regardless that HNSCCs were FA-proficient or FA-deficient, all tested samples expressed high levels of ErbB1 and ErbB2, with ErbB1 expression being the highest of all ErbB members (Figure 1). This membrane expression can also be observed in the immunohistochemistry analysis showed in VU-1131 tumor xenografts in Figure S1. ErbB3 and ErbB4 were expressed in a much lower proportion of HNSCC cell lines and at much lower levels. These findings indicate that EbrbB1 and ErbB2 receptors should constitute good CAR-T cell targets for the treatment of HNSCC from both the general population and from patients with FA.

### Efficient generation of panErbB CAR-T cells from peripheral blood of HDs and patients with FA

Considering that the generation of CAR-T cells requires a significant proliferation stress on donor T cells, and given that several molecular and cellular alterations have been reported for FA cells, we first investigated the feasibility of generating functionally competent CAR-T cells from patients with FA, as compared to HDs.

**Figure 2:**
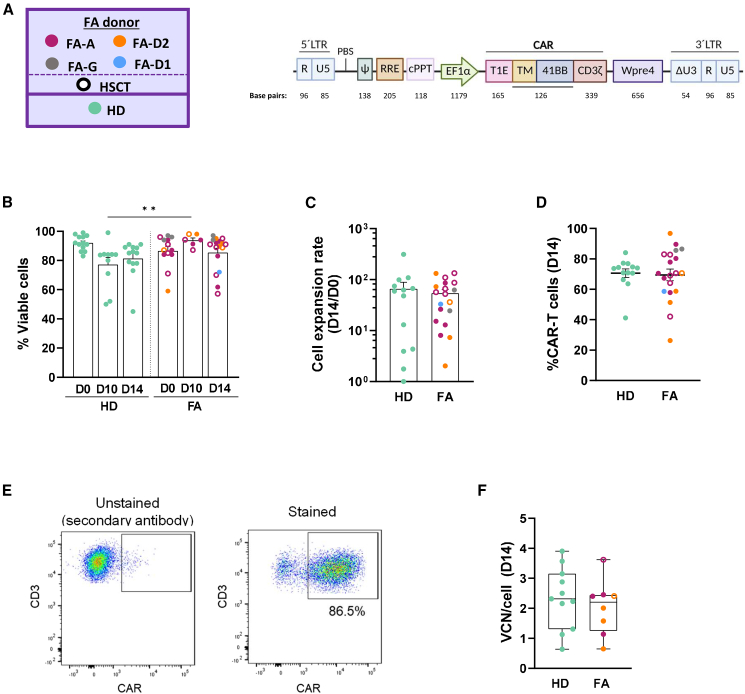
Efficient generation of lentiviral-mediated panErbB CAR-T cells from HDs and patients with FA (A) Schematic diagram depicting the panErbB CAR LV constructed to generate panErbB CAR-T cells. (B), (C), (D), and (E) represent the analysis of the main parameters defining the CAR-T cell production from PB samples obtained from HDs and patients with FA, according to the optimized experimental conditions shown in Figure S2. Each dot represents data corresponding to CAR-T cells generated from a single individual. HDs are represented in green. The four FA complementation groups are represented by different colors. Filled dots and open dots correspond, respectively, to untransplanted and transplanted FA individuals. (B) Cell viability of HD and FA CAR-T cells at day 0, 10, and 14 of the manufacturing protocol. (C) Cell expansion rate of HD and FA CAR-T cells. (D) Percentage of CAR-T cells at the end of the manufacturing process of HD and FA PB samples. (E) Representative example of the flow cytometry showing the gating strategy for evaluating the percentage of CAR^+^ cells (FA-772). (F) VCN/cell obtained at day 14 of the manufacturing protocol in HD and FA CAR-T cells. Bars in (B), (C), and (E) show the mean ± SEM. In (D), bars show the min to max plot of the VCN/cell. Statistical analyses were performed following Mann-Whitney or Student’s *t* test after Saphiro-Wilk’s test for unpaired comparisons. ∗∗*p* < 0.01. FA-A, FA-G, FA-D1, and FA-D2, FA CAR-T cells generated from patients with mutations in *FANCA*, *FANCG*, *BRCA2*, and *FANCD2*, respectively. HSCT, cells generated from a transplanted FA patient; HD, healthy donor. 5′ LTR (RU5) region; PBS, primer-binding site; Ψ, packaging signal; RRE, rev-response element; cPPT, central polypurine tract; EF1α, elongation factor 1α promoter; CAR, codon-optimized version of the CAR (with T1E as extracellular domain, TM as CD28 transmembrane domain, 41BB as co-stimulatory domain, and CD3 ζ as T cell-activating domain); Wpre4 (mutated Wpre, woodchuck hepatitis virus post-transcriptional regulatory element as a stabilization element).

The CAR designed to target ErbB receptors for sporadic and FA HNSCCs carries the panErbB-specific T1E extracellular domain,^31^ fused to the 4-1BB co-stimulatory domain and the CD3ζ-activating domain, and was placed under the regulation of the elongation factor alpha promoter in an LV, as shown in Figure 2A. To optimize T cell transduction with panErbB CAR LVs, peripheral blood mononuclear cells (PBMNCs) from HDs and patients with FA were pre-stimulated, transduced with different multiplicities of infection (MOIs) of the LV, and expanded for 10 days in the presence of interleukin (IL)-7 and IL-15. As shown in Figure S2, the proportion of CAR-T cells reached a plateau at around 80% of CAR-positive cells when MOIs of at least 10 infective transduction units per cell (TU/cell) were used. Under these conditions, vector copy numbers per cell (VCNs/cell) were in the range of 2–4 (Figure S2). Almost identical results were obtained when PBMNCs from HDs and patients with FA were tested (see Figures S2A and S2B, respectively). Based on these observations, an MOI of 10 TU/cell was used throughout.

Data in Figures 2B–2F show the optimized generation of panErbB CAR-T cells derived from HDs and patients with FA, after incubation under hypoxic conditions in G-Rex GMP-scalable devices (WilsonWolf).^32^ Mean viabilities higher than 70% (Figure 2B), expansion rates higher than 50-fold (Figure 2C), and percentages of CAR-T cells around 75% (see Figure 2D and representative analyses in Figure 2E; details of the staining procedure are shown in materials and methods) were obtained. Additionally, mean VCNs/cell of around 2 copies were observed in these samples (Figure 2F), highly compatible with the clinical use of these cells. All these parameters were similar when CAR-T cells were generated from HDs and from patients with FA, regardless of their complementation group and whether patients had been previously transplanted or not (represented by different symbols in Figures 2D–2F).

**Figure 3:**
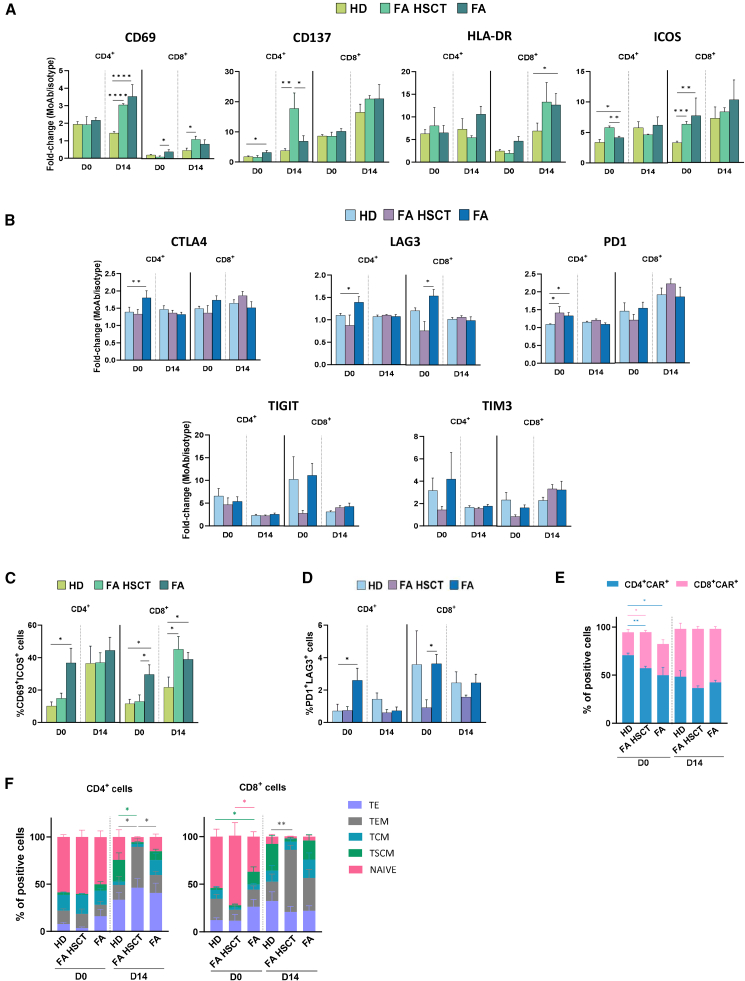
Characterization of the immunophenotype of panErbB CAR-T cells from HDs and patients with FA All phenotypes were studied in CAR^+^ and for both CD4^+^ and CD8^+^ cells. (A) Analysis of activation markers: CD69, CD137, HLA-DR, and ICOS. (B) Analysis of exhaustion markers: CTLA4, LAG3, PD1, TIGIT, and TIM3. (C) Percentage of cells that co-express CD69 and ICOS markers as additional activation markers. (D) Percentage of cells co-expressing PD1 and LAG3 as additional exhaustion markers. In all instances, flow cytometry analyses were measured on day 0 and day 14 of the manufacturing protocol in HD and FA samples. The expression is the MFI fold-change for each specific marker relative to the MFI of its corresponding isotype control. All bars in (A), (B), (C), and (D) show the mean ± SEM: HD (*n* = 9); FA HSCT (*n* = 4); FA (*n* = 9). (E) Proportion of CD4^+^CAR^+^ and CD8^+^CAR^+^ cells. Bars show the mean ± SEM: HD (*n* = 8); FA HSCT (*n* = 4); FA (*n* = 6). (F) Analysis of differentiation subpopulations: TCM, TE, TEM, NAIVE, and TSCM cells at day 0 and 14. Bars show the mean ± SEM: HD (*n* = 8); FA HSCT (*n* = 4); FA (*n* = 9). Statistical analyses were performed following Mann-Whitney or Student’s *t* test after Saphiro-Wilk’s test for unpaired comparisons. ∗*p* < 0.05; ∗∗*p* < 0.01; ∗∗∗*p* < 0.001; ∗∗∗∗*p* < 0.0001. TE, T effector cells; TEM, T effector memory cells; TCM, T central memory cells; TSCM, T stem central memory cells; NAIVE, naive T cells.

The balance among the activation, exhaustion, and differentiation status of CAR-T cells and also between CD4 and CD8 CAR-T cell subpopulations determines the therapeutic efficacy of these cells. Therefore, the expression levels of four commonly tested activation markers (CD69, CD137, HLA-DR, and ICOS)^33^^,^^34^ were assessed in CD4 and CD8 subpopulations at the onset and upon the completion of the manufacturing of CAR-T cells from HDs and from patients with FA (Figure 3A; samples from both transplanted and not transplanted—none of them mosaic patients—were used in these studies). In parallel, the exhaustion status of these CAR-T cells deduced from the expression of CTLA4, LAG3, PD1, TIGIT, and TIM3^35^^,^^36^ was analyzed (Figure 3B). To have a more accurate phenotype profile of these cells, the percentage of panErbB CAR-T cells co-expressing two key activation markers, CD69 and ICOS, and two exhaustion markers, PD1 and LAG3, is presented in Figures 3C and 3D, respectively. Data shown in Figures 3A and 3C revealed no differences in the expression of activation markers between HD and FA CAR-T cells at the end of the manufacturing process, regardless of whether patients with FA had been transplanted or not. We also observed low expression levels of exhaustion markers by these cells, with minimal differences between HDs and the different types of FA samples (Figures 3B and 3D). Also, when the CD4/CD8 CAR-T cell ratio was determined, an increased ratio was observed in all instances with respect to values determined on day 0, and again, similar results were observed between samples corresponding to HDs and patients with FA (Figure 3E). These findings suggest that panErbB CAR-T cells generated from HDs or patients with FA, either transplanted or not, exhibit activation profiles without significant signs of exhaustion and with a balanced proportion of CD4^+^ and CD8^+^ cells.

Given that stemness and memory-related phenotypes are critical for the long-term efficacy of CAR-T cells, we analyzed potential differentiation changes in CD4^+^ and CD8^+^ CAR-T cells generated from HDs and patients with FA (Figure 3F). We focused on five distinct subpopulations: effector T cells, effector memory T cells (TEM), central memory T cells (TCM), stem-central memory T cells (TSCM), and naive T cells (NAIVE), by means of the analysis of the CCR7, CD45-RA, and CXCR3 markers (Figure S3).^37^^,^^38^ As expected, a reduction in the proportion of naive cells took place from day 0 to the final CAR-T cell product in both CD4^+^ and CD8^+^ populations, leading to a more effector-dominant phenotype. A higher proportion of TSCM cells was observed in CAR-T cells from HDs compared to patients with FA, either transplanted or not, while a higher percentage of TEM cells was observed in CD4^+^ and CD8^+^ CAR-T cells corresponding to transplanted FA patients compared to untransplanted patients or HDs (see Figure 3F and representative analyses in Figure S3).

### *In vitro* cytotoxic effects of HD and FA panErbB CAR-T cells against HNSCCs and healthy cells

**Figure 4:**
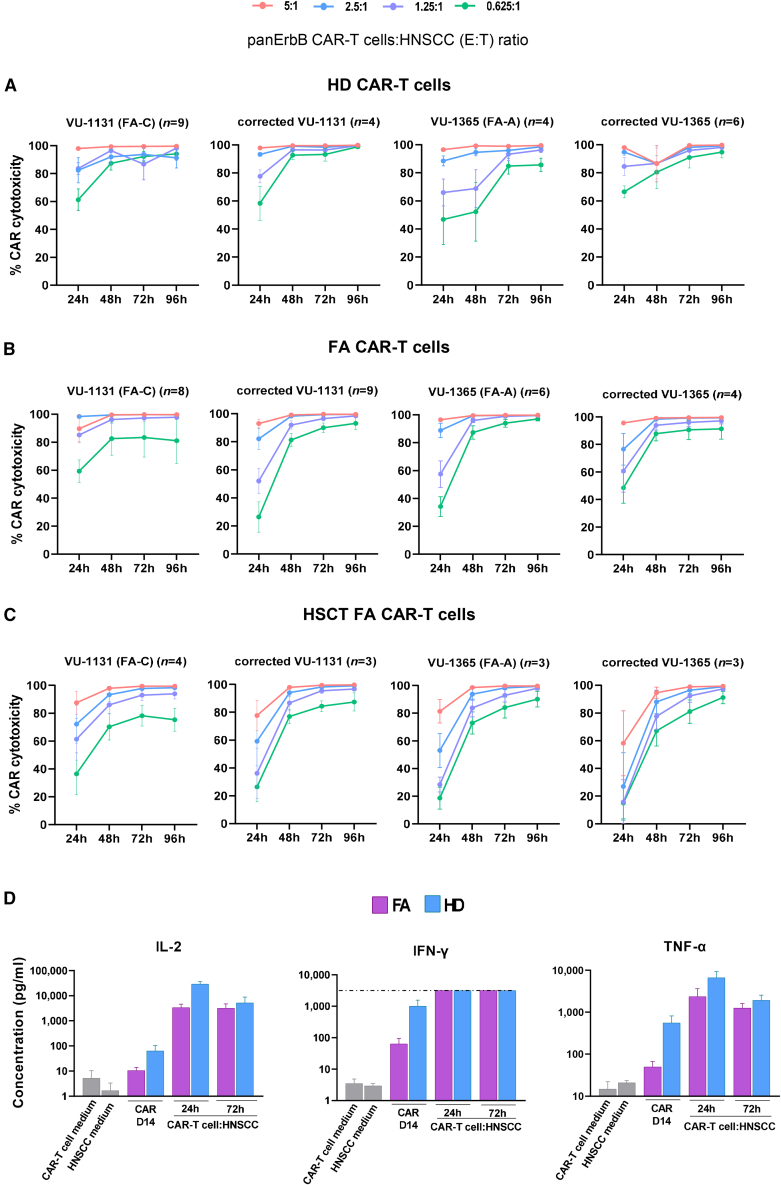
*In vitro* cytotoxicity of FA and HD panErbB CAR-T cells against HNSCC cell lines (A) Cytotoxicity of HD CAR-T cells co-cultured with VU-1131 (FA-C-deficient cell line) and its retroviral corrected version (VU-1131+FANCC); VU-1365 (FA-A-deficient cell line) and its retroviral corrected version (VU-1365+FANCA) at different E:T ratios. (B) Cytotoxicity of CAR-T cells derived from untransplanted patients with FA against the same HNSCC cell lines shown in (A). (C) Cytotoxicity of CAR-T cells from transplanted FA patients against the same HNSCC cell lines used in (A) and (B). In (A), (B), and (C), each dot represents the mean ± SEM of different samples (*n* indicated at the top of each graph). (D) Analysis of different cytokine production by panErbB CAR-T cells prior to and after incubation with HNSCCs. IL-2, IFN-γ, and TNF-α were quantified in FA patients and HD samples by flow cytometry bead arrays. IFN-γ levels in co-cultured samples were above the upper detection level of the assay, shown by a dotted line. Bars show the mean ± SEM: HD at day 14 and 24 h of co-culture (*n* = 7), HD samples at 72 h of co-culture (*n* = 5), FA samples at day 14 (*n* = 9), FA samples at 24 h of co-culture (*n* = 6), and FA samples at 72 h of co-culture (*n* = 8). Statistical analyses were performed following Mann-Whitney or Student’s *t* test after Shapiro-Wilk’s test for unpaired comparisons. Two outliers were excluded for the IL-2 analysis, one for the IFN-γ analysis and one for the TNF-α analysis, using the ROUT method (Q = 2%).

In these experiments, the *in vitro* cytotoxicity of HD and FA panErbB CAR-T cells was evaluated both against FA-proficient and FA-deficient HNSCC cell lines, using four different effector:target (E:T) ratios. The results shown in Figures 4A–4C reveal a potent and similar cytotoxic activity of FA CAR-T cells—either derived from untransplanted or transplanted FA patients—compared with CAR-T cells generated from HDs. To evaluate the non-specific killing of panErbB CAR-T cells against these tumor cell lines, HNSCC co-cultures were established either with panErbB CAR-T cells or with T lymphocytes that had been transduced with an EGFP-LV (EGFP^+^ T cells) using a 1.25:1 ratio. The results shown in Figure S4 confirmed the specific cytotoxicity of panErbB CAR-T cells, allowing us to conclude that panErbB CAR-T cells generated from HDs or patients with FA exhibit a potent *in vitro* cytotoxic capacity against multiple HNSCC cell lines, either proficient or deficient in the FA pathway.

To further assess the functionality of panErbB CAR-T cells, we quantified the secretion profile of three key cytokines: IL-2, interferon-gamma (IFN-γ), and tumor necrosis factor alpha (TNF-α) on day 14 of the CAR-T cell manufacturing process, as well as 24 and 72 h after co-culture with HNSCCs (VU-1131). This FA cancer cell line, previously reported to exhibit rapid growth in xenograft models, was selected for cytokine release studies and for subsequent *in vivo* CAR-T cell cytotoxicity evaluations.^39^ As additional controls, conditioned media from HNSCCs and from CAR-T cells that had been cultured individually were used to determine baseline cytokine levels. The results summarized in Figure 4D revealed marked increases of these cytokines once the CAR-T cells were co-incubated with HNSCCs. Additionally, similar secretion kinetics, though higher cytokine levels were observed in HD compared to FA CAR-T cells.

Taking into account that ErbB receptors can also be expressed in healthy epithelial cells, we also evaluated the expression of these molecules in primary human oral fibroblasts and keratinocytes and investigated the cytotoxic effects of panErbB CAR-T cells on these cells. As expected, these primary cells expressed high levels of both ErbB1 and ErbB2 (Figure S5). Additionally, we confirmed the cytotoxic effect of panErbB CAR-T cells against these primary cells (Figure S5B). Considering the results obtained in primary fibroblasts, we performed similar analyses in cancer-associated fibroblasts (CAFs), with known tumor promotion effects. The results shown in Figure S5C evidenced the high expression of ErbB1 and ErbB2 receptors in these cells, as well as the cytotoxic effect of panErbB CAR-T cells against these CAFs.

### *In vivo* antitumor effects of HD and FA panErbB CAR-T cells against HNSCCs implanted into immunodeficient mice

In subsequent experiments, panErbB CAR-T cells generated from HDs and patients with FA were inoculated into HNSCC tumors previously implanted (5 × 10^6^ tumor cells of VU-1131 per flank) into immunodeficient NSG mice.^29^ Mice were euthanized when tumor burdens exceeded 3 × 10^10^ to 8 × 10^10^ p/s or when necrotic areas were observed in the tumor areas. In some experiments, mice treated with CAR-T cells, thus showing lower bioluminescence signals, were also euthanized at the same post-implantation times to analyze in parallel, levels of hCD3^+^, EGFP^−^, and CAR-T cell infiltration in all animal groups.

**Figure 5:**
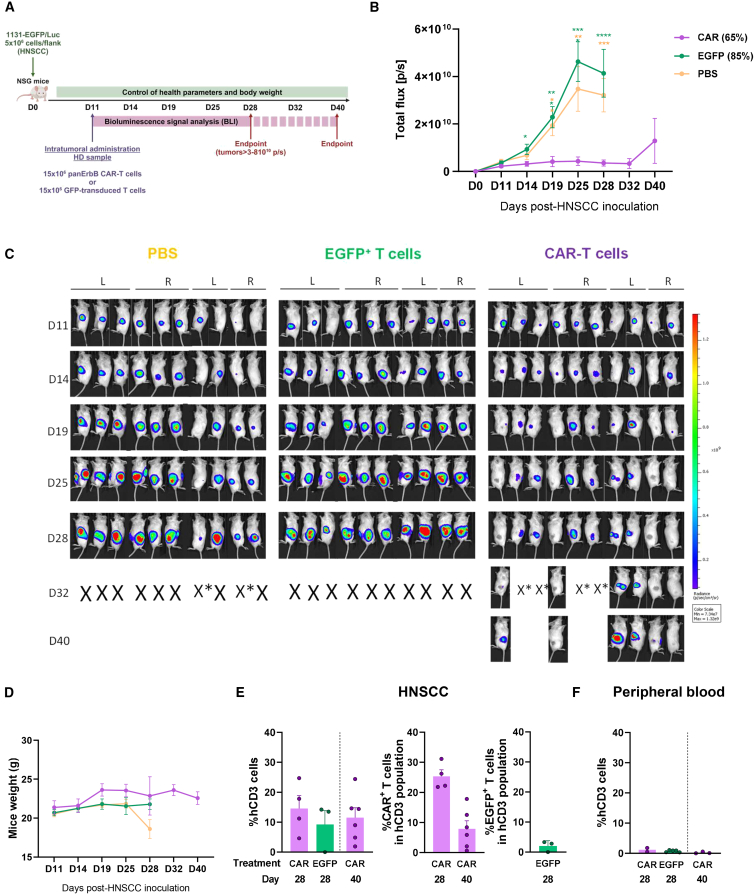
*In vivo* antitumor effects of HD panErbB CAR-T cells against HNSCC xenografts implanted into immunodeficient mice (A) Schematic representation of the experimental protocol used to examine the *in vivo* antitumoral effects of panErbB CAR-T cells. Mice were inoculated with VU-1131+GFP/Luc tumor cells, and 11 days later, tumors were injected with a single dose of CAR-T cells (purple), EGFP-T lymphocytes (green), or PBS (yellow) from a single HD sample. (B) Mean ± SEM of tumor burden was assessed as normalized BLI intensity for each group (*n* = 10 tumors per group; 2 tumors per mouse). Statistical differences between the different CAR-T cell-treated groups on day 19 post tumor inoculation are shown on the right. (C) IVIS bioluminescence imaging of individual mice showing tumor burden in both left and right flanks, captured at the indicated time points. X represents mice euthanized due to excessive tumor burden; *mice marked with* “*X∗*” were sacrificed at the same time point to enable comparative analysis of tumor infiltrates. L, left flank; R, right flank. (D) Weight analysis of mice at different time points, presented as the mean for each group ± SEM (*n* = 5). (E) Presence of hCD3^+^ cells and hCD3^+^CAR^+^ cells in tumor infiltrates on days 28 and 40 after tumor implantation. Bars show the mean ± SEM in the CAR-T-treated group (*n* = 4 on day 28; *n* = 6 on day 40) and the EGFP-treated group (*n* = 3, day 28). (F) Presence of hCD3^+^ cells in PB on days 28 and 40 after tumor implantation. Bars show the mean ± SEM in CAR-treated group at day 28 (*n* = 2), mice in GFP-treated group at day 28 (*n* = 5), mice in CAR-treated group at day 40 (*n* = 3). Statistical analyses were performed following Mann-Whitney or Student’s *t* test after Saphiro-Wilk’s test for unpaired comparisons. ∗∗∗*p* < 0.001; ∗∗∗∗*p* < 0.0001. CAR, PanErbB CAR-T cells; EGFP, T cells transduced with the EGFP LV.

As illustrated in the experimental protocol shown in Figure 5A, once the tumors were clearly visible (day 11 post VU-1131 injection), a single dose of 15 million of the HD CAR-T cell manufacturing product (65% of CAR expression)—or an equivalent number of EGFP-transduced T cells (85% EGFP^+^ cells) generated from the same peripheral blood (PB) sample—was administered into tumors implanted in both flanks of recipient mice. Mean bioluminescence imaging (BLI) results and individual images are represented in Figures 5B and 5C, respectively. These figures revealed the rapid tumor growth in the control groups in which tumors were treated with PBS or EGFP^+^ T cells. In marked contrast with this observation, the intratumoral inoculation of panErbB CAR-T cells controlled the tumor growth in most animals for a significant period of time; and in 3 out of 10 treated tumors, no bioluminescence signals were observed throughout the observation period. In several cases, the tumor remission was associated with the apparition of local scars (see local gray areas in Figure 5C), probably due to local inflammatory responses against the tumor area. The intratumoral administration of panErbB CAR-T cells did not result in body weight loss (Figure 5D) or illness signs (e.g., ruffled hair or respiratory distress; not shown).

Flow cytometry analyses of tumors that persisted for 28–40 days after HNSCC inoculation showed the presence of human CD3^+^ cell infiltrates either in tumors that had been treated with CAR-T cells or EGFP-T cells. The proportion of CAR-T cells determined on day 40 was markedly lower compared with values determined on day 28, possibly due to CAR-T cell stress or exhaustion (Figure 5E). In three tumors treated with control EGFP-transduced T cells, the presence of EGFP^+^ cells on day 28 was even lower (Figure 5E), suggesting the very limited proliferation ability of these cells in the tumors. In all instances, the proportion of human T cells detected in PB of mice treated with CAR-T or EGFP-T cells was extremely low (Figure 5F), indicating minimal migration of these cells to systemic circulation.

**Figure 6:**
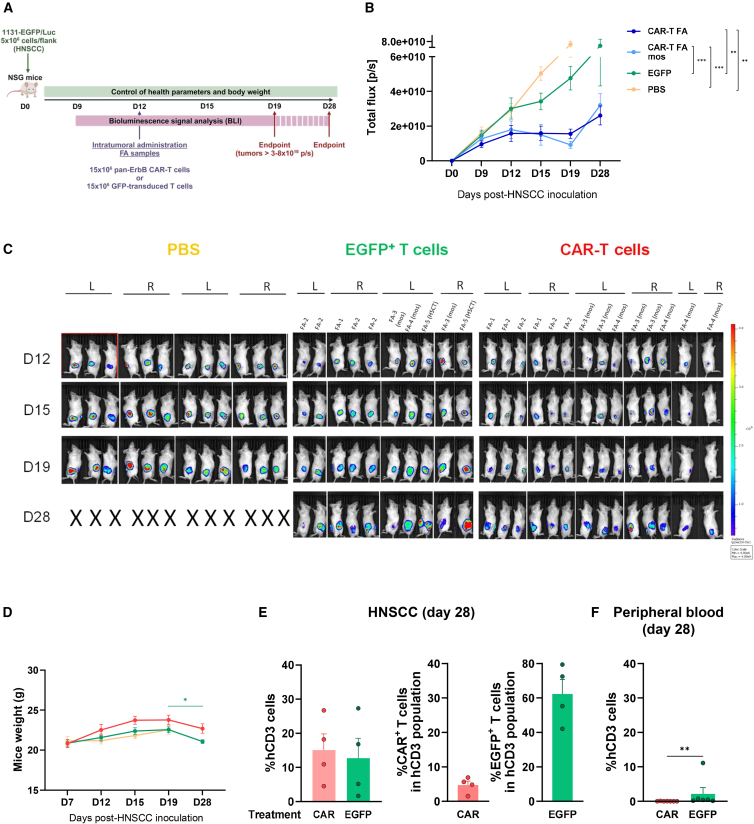
*In vivo* antitumor effects of panErbB CAR-T cells derived from patients with FA (A) Schematic representation of the experimental protocol used to examine the *in vivo* anti-HNSCC effects of FA panErbB CAR-T cells. Mice were inoculated with VU-1131+GFP/Luc tumor cells, and 12 days later, tumors were injected with a single dose of CAR-T cells from patients with FA. (B) Mean ± SEM of tumor burden was assessed as normalized BLI intensity for each FA-CAR T cell group: standard FA patients (not transplanted, not mosaic; dark blue) and mosaic FA patients (mos; in light blue). Controls consisted of EGFP-T lymphocytes (green) and PBS (yellow). (C) IVIS bioluminescence imaging of individual mice showing tumor burden in both left and right flanks, captured at the indicated time points. X: mice euthanized due to the tumor burden; L, left flank; R, right flank. (D) Weight analysis of mice at different time points is presented as the mean ± SEM: unified FA CAR-T cell-treated group (*n* = 7; red line), EGFP (*n* = 6; green), and PBS (*n* = 6; yellow). (E) Presence of hCD3^+^ cells and hCD3^+^CAR^+^ cells in tumor infiltrates on day 28. Bars show the mean ± SEM (*n* = 4). Symbols as in (D). (F) Presence of hCD3^+^ cells in PB on day 28. Bars show the mean ± SEM: CAR-treated group (*n* = 7), GFP (*n* = 6). Symbols as in (D). Statistical analyses were performed following Mann-Whitney or Student’s *t* test after Saphiro-Wilk’s test for unpaired comparisons and represented in the legend relative to day 19. ∗∗*p* < 0.01; ∗∗∗*p* < 0.001.

A similar experiment was conducted using FA panErbB CAR-T cells (see experimental protocol in Figure 6A). In this experiment, PB samples from two standard patients (not transplanted and not mosaic patients), three mosaic patients, and one transplanted patient were used (see patient codes in Figures 6B and 6C). The response of CAR-T cells derived from standard FA patients—with a non-functional FA pathway—was represented differentially (dark blue line in Figure 6B) from samples corresponding to transplanted and mosaic patients—both with an expected functional FA pathway (see light blue line in Figure 6B). Percentages of CAR-T cells and EGFP-T cells obtained in these experiments ranged from 77% to 91% and from 22% to 96%, respectively.

In this experiment, a very fast growth of HNSCC tumors that had been treated with PBS or EGFP-T cells was observed. Nevertheless, the intratumoral inoculation of either type of FA CAR-T cells efficiently controlled the tumor growth in NSG immunodeficient mice until day 19 post-HNSCC implantation (Figures 6B and 6C). Since no differences in the antitumor effects mediated by CAR-T cells from either type of FA patient were observed, the results of all FA CAR-T cell types were unified in subsequent panels (see red lines and symbols in Figures 6D–6F). As happened with HD CAR-T cells, the treatment with FA CAR-T cells was associated with higher body weights in these animals, as compared to mice treated with PBS or EGFP-T cells (Figure 6D). Flow cytometry analyses of tumor infiltrates revealed a similar proportion of human CD3^+^ cells in tumors that had been treated either with CAR-T cells or EGFP-T cells (Figure 6E, left), although a lower proportion of CAR-T cells was found compared to the proportion of EGFP^+^ cells corresponding to the EGFP-T-treated mice (Figure 6E, right). As observed with HD samples, almost no CAR-T cells or EGFP-T cells were detected in the PB of these mice (Figure 6F), confirming the minimal systemic dissemination of these cells.

**Figure 7:**
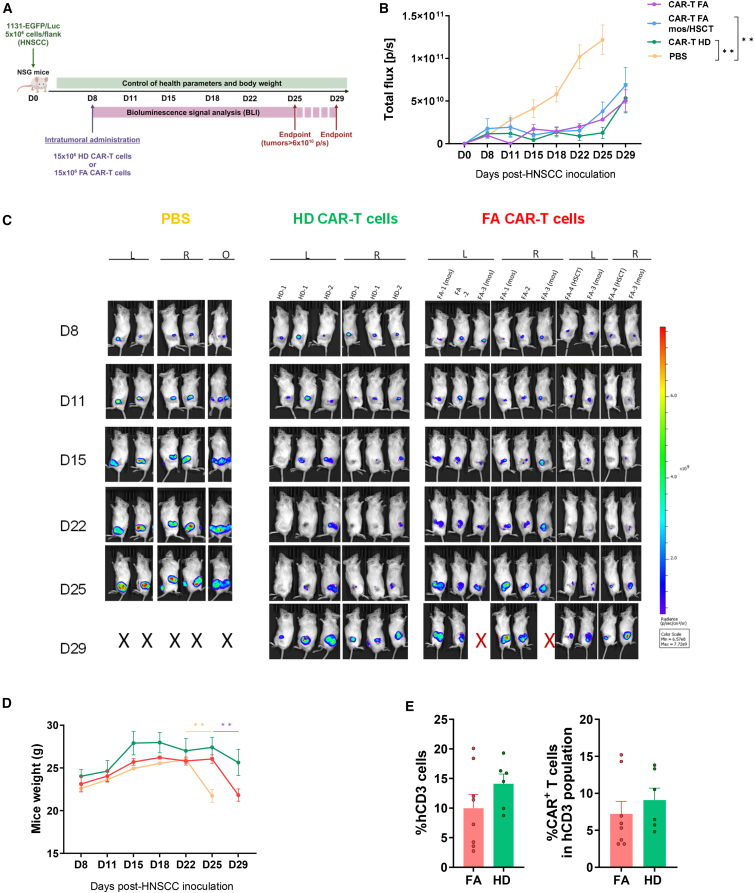
Comparison of the *in vivo* antitumor effects of panErbB CAR-T cells derived from HDs and patients with FA (A) Schematic representation of the experimental protocol used to examine in parallel the *in vivo* anti-HNSCC effects of HD and FA panErbB CAR-T cells. Mice were inoculated with VU-1131+GFP/Luc tumor cells, and 8 days later, tumors were injected with CAR-T cells from HDs or patients with FA, or with EGFP-T cells or PBS as controls; (B) Mean ± SEM of tumor burden was assessed as normalized BLI intensity for each group: CAR-T cells from HDs (purple), standard FA patients (dark blue), transplanted or mosaic FA patients (HSCT/mos, light blue), and PBS (yellow). (C) IVIS bioluminescence imaging of individual mice showing tumor burden in both left and right flanks, captured at the indicated time points. Overhead images of the mouse in the fifth column of the PBS group are shown highlighting the presence of three distinct tumor masses. X, mice euthanized due to the tumor burden; X in red color related to mice that died during the experiment. L, left flank; R, right flank; O, overhead image. (D) Weight analysis of mice at different time points is presented as the mean ± SEM: FA CAR-treated group (*n* = 5; red line), HD CAR-treated group (*n* = 3; line), and PBS (*n* = 3; yellow line). (E) Presence of hCD3^+^ cells and hCD3^+^CAR^+^ cells in tumor infiltrates on day 29. Bars show the mean ± SEM: FA CAR-treated group (*n* = 8) and HD CAR-treated group (*n* = 6). Symbols as in (D). Statistical analyses were performed following Mann-Whitney or Student’s *t* test after Saphiro-Wilk’s test for unpaired comparisons and represented in the legend relative to day 19. ∗∗*p* < 0.01.

In a final *in vivo* experiment, the efficacy of CAR-T cells derived from HDs and patients with FA was investigated in parallel (Figure 7A). Two samples from HDs were used to treat a total of 6 tumors. Additionally, one sample from a standard FA patient, one sample from a transplanted patient, and two samples from mosaic patients were used to treat a total of 10 tumors. Percentages of CAR-T cells ranged from 72% to 85%. As controls, 6 tumors were inoculated with PBS. Consistent with data from Figures 5 and 6, a single intratumoral administration of HD and FA CAR-T cells—regardless of whether donor FA patients were standard, mosaic, or had been subject to hematopoietic transplantation—efficiently controlled tumor progression up to day 25 post-HNSCC implantation (Figures 7B, 7C, and S6B). Similar to HD CAR-T cells, FA CAR-T cell-treated mice progressed with higher body weights compared to the PBS-treated group (Figure 7D). Flow cytometry analyses of tumor-infiltrating cells also revealed a similar proportion of human CD3^+^ cells in FA and HD CAR-T cell-treated tumors, with no significant differences in CAR-T levels (Figure 7E). The presence of CAR-T cells in PB was undetectable in mice treated in this experiment, reinforcing the notion of minimal systemic dissemination of intratumorally administered CAR-T cells.

This final set of experiments demonstrate the *in vivo* anti-HNSCC effect associated with the intratumoral administration of panErbB CAR-T cells, either generated from HDs or from transplanted or untransplanted patients with FA, with no significant off-tumor toxicity effects.

## Discussion

CAR-T cell therapy has shown a remarkable clinical efficacy, particularly in patients with B cell and plasma cell malignancies.^20^ Although the efficacy of CAR-T cells in solid tumors remains challenging, recent advances suggest that CAR-T cell therapies will also achieve a significant clinical impact.^40^^,^^41^ While ideal anticancer therapies would be based on the targeting of specific cancer antigens, clinical data obtained in patients with hematological malignancies have shown that the highest efficacy of CAR-T cells has been achieved by means of the targeting of cell lineage-associated antigens, such as those expressed in the B cell lineage. Additionally, the use of dual CAR-T cells targeting two different B cell antigens (e.g., CD19 and CD20) has shown improved efficacy to prevent the relapse of malignant B cells that have lost expression of one of the targeted antigens.^42^ Also in the case of solid tumors, including hepatocellular carcinoma, cervical, glioblastoma, and ovarian cancers, preclinical as well as early clinical data have shown the reduced tumor escape of dual CAR-T cells in comparison with CAR-T cells targeting a single antigen.^43^^,^^44^

Several ErbB-targeted therapies are currently under investigation for HNSCC treatment, including monoclonal antibodies or antibody-drug conjugates (e.g., cetuximab targeting EGFR and trastuzumab targeting HER2) or small-molecule tyrosine kinase inhibitors such as erlotinib for EGFR and lapatinib for EGFR and HER2, which usually require repeated administration. Each of these therapies have demonstrated varying degrees of clinical efficacy depending on the tumor type and patient context and showed limitations including acquired resistance, incomplete tumor eradication, and off-target toxicities.^45^^,^^46^^,^^47^ In the particular case of FA, a clinical trial for patients with HNSCC has recently been initiated with the non-genotoxic EGFR signaling inhibitor, afatinib (AFAN trial, EUCT no.: 2024-511477-29-00).^48^

Compared to pharmacological therapies, CAR-T cells have shown tumor cell killing even in cases with low antigen density, via multiple mechanisms, including direct tumor cell killing by granzymes and perforins and cytokine release, which can recruit other immune cells.^49^^,^^50^ Aiming at improving the efficacy of CAR-T cells in solid tumors, these cells have been combined with other treatments such as immunomodulation or radio-chemotherapy,^51^ although the use of these therapies should be carefully considered in patients with FA due to their heightened sensitivity to DNA damage and genotoxic stress.

In the particular case of HNSCC, a previous phase 1 clinical trial was already conducted, in which the intratumoral administration of CAR-T cells expressing a panErbB ligand—T1E28z CAR-T cells—was given. Interim results of this clinical trial showed that this treatment was safe and mediated a significant clinical efficacy in patients with advanced and/or recurrent HNSCCs.^30^ To further improve the safety of this CAR, here we replaced the gamma-RV used in the previous trial by an LV. Additionally, the IL-4/IL-15 receptor sequence previously used to facilitate the *ex vivo* expansion of the CAR-T cells was deleted due to current improvements in CAR-T cell manufacturing. In addition, the CD28 intracellular signal of the CAR was replaced by the 4-1BB sequence, which has been associated with a higher proportion of TCM cells and with a longer persistence of the CAR-T cells in the patient.^52^^,^^53^^,^^54^ The fact that panErbB CAR-T cells generated in our study use a clinically applicable LV and that the production of these CAR-T cells was made using a GMP-scalable manufacturing process reveals the translational impact of our study.

Our results firstly show that all tested HNSCC cell lines—regardless of whether they had a functional FA pathway or not—expressed high levels of ErbB1 (EGFR) and ErbB2 in their cell membrane. This observation and recent findings showing significant EGFR copy-number amplification in primary FA HNSCC cells^22^ strongly suggest that the panErbB CAR-T cells generated in this study should efficiently target HNSCCs in non-FA as well as in patients with FA. Taking into account that panErbB CAR-T cells behave as multi-antigen-specific CAR-T cells, capable of recognizing eight different hetero- and homodimers generated by ErbB receptors, this indicates that the possibility of HNSCC immune escape after treatment with these CAR-T cells should be much lower compared with CAR-T cells targeting a single receptor. Additionally, the fact that FA is the disease with the highest incidence of HNSCCs^8^^,^^10^^,^^55^ and that standard treatments for HNSCC in patients with FA are of very limited efficacy^18^ reinforces the relevance of panErbB CAR-T cell therapy in these patients.

At the initiation of these studies, we speculated that due to the molecular and biochemical abnormalities characteristic of FA cells,^4^^,^^5^^,^^6^^,^^7^ the generation of functional CAR-T cells with significant antitumoral activity could be limited when these cells are generated from patients with FA. Thus, we first conducted a comprehensive set of experiments aiming to compare the feasibility of generating functionally competent CAR-T cells from HDs and patients with FA. Consistent with previous findings showing that FA T lymphocytes can be efficiently activated and expanded *in vitro*,^56^^,^^57^ we demonstrated that panErbB CAR-T cells could be efficiently generated not only from HDs but also from FA patients—either transplanted or not—and corresponding any of the four different complementation groups tested in our experiments. These results strongly support the feasibility of manufacturing CAR-T cells from all FA patient types, even without the necessity of correcting their genetic defect. Both HD and FA-derived panErbB CAR-T cells showed balanced CD4^+^/CD8^+^ percentages and similar phenotypes in both cell populations.^58^ In addition, a robust and similar cytotoxicity against HNSCCs was also observed not only *in vitro* but also following the direct administration of panErbB CAR-T cells into HNSCCs previously implanted into immunodeficient NSG mice, demonstrating their antitumor effects, regardless of the donor origin. As expected from a single administration of human CAR-T cells into immunodeficient mice, the marked inhibitory effects of these cells against implanted tumors were frequently followed by relapses in the long term.^59^ In clinical practice, however, escalating numbers of CAR-T cells and even serial CAR-T cell treatments could be considered. Additionally, secondary immune responses following treatment with autologous CAR-T cells into immunocompetent patients could be expected, which would further improve the antitumoral efficacy of the therapy.^60^^,^^61^ Aiming to evaluate the efficacy of panErbB CAR-T cells in syngeneic FA mice with spontaneous HNSCCs, new studies will be conducted in FA mouse models that recapitulate the generation of HNSCCs in patients with FA.^62^

The intratumoral delivery of CAR-T cells could be beneficial in patients with primary, locally advanced unresectable tumors and also for preventing tumor progression when detected prior to surgical interventions that could result in severe oropharyngeal sequelae.^30^^,^^63^ The proposed therapy could be applicable to the general population of patients with HNSCCs, since the survival rates of patients with HNSCC remain suboptimal and surgical interventions often lead to severe functional impairments.^18^ Nevertheless, its use in FA patients with HNSCCs may constitute a remarkable novel approach, given the extreme sensitivity of these patients to chemo- and radiotherapy.

While the expression of ErbB members in healthy cells, such as fibroblasts and epithelial cells, should prevent the systemic administration of panErbB CAR-T cells in human subjects, the therapeutic efficacy associated to the intratumoral administration of these cells may be benefited from their interaction with cells of the tumor microenvironment, such as the CAFs (see Figure S5).^64^^,^^65^

In addition to the obvious cost advantage associated to the manufacturing of low numbers of CAR-T cells to be administered intratumorally, compared with numbers required for systemic administrations,^66^ the possibility of cryopreserving multiple CAR-T cell bags for subsequent re-administrations constitutes an additional significant advantage associated to intratumoral CAR-T cell therapies. Additionally, the generation and cryopreservation of T cell receptor and HLA-edited allogeneic CAR-T cells from HDs would serve for the immediate administrations in patients.^67^ Finally, the development of fourth-generation panErbB CAR-T cells co-expressing enhancer molecules such as IL-15 or IL-18 would constitute more potent anti-HNSCC therapies.^68^

Taken together, our study demonstrates for the first time the feasibility of generating CAR-T cells from patients with an inherited genetic disorder such as FA, in this particular case for the non-genotoxic treatment of HNSCCs. Additionally, our study suggests that other CAR-T cell types could be generated from patients with FA for the treatment of other malignancies highly prevalent in this disease, such as acute myeloid leukemia.

## Materials and methods

### PB samples from HDs and patients with FA

PB samples from HDs (ages 19–60 years) were collected in EDTA tubes and were provided by the CIEMAT Health Department, after approval by the Ethics Committee of the Fundación Jiménez Díaz (PIC134-19) and signature of the corresponding informed consents. PB samples from patients with FA (ages 2–56 years) of either transplanted or untransplanted patients, including patients with corrected cells in PB due to mosaicism, were obtained following approval by the Ethic Committee of the Hospital Fundación Jiménez Díaz in Madrid (PIC291-24 CIEMAT). PBMNCs were obtained by Ficoll-Plaque PLUS (GE Healthcare) density gradient centrifugation followed by cell washing in PBS.

### HNSCC cell lines and primary cells

HNSCC cell lines derived from FA patients’ mouth mucosa were VU-1131 (mutated in *FANCC*) and VU-1365 (mutated in *FANCA*), kindly provided by Dr. J Dorsman (Amsterdam UMC, Holland). The corrected versions of VU-1131 and VU-1365 cell lines were generated in our laboratory by transduction with gamma-RVs carrying the wild-type versions of *FANCC* or *FANCA* genes, as previously described.^56^ CAL27 and CAL33 cell lines derived from squamous tongue tumors were kindly provided by Dr. Silvio Gutkind (UC San Diego, CA, USA). FA-A versions of these cell lines were generated by means of the knockout of *FANCA* using the CRISPR-Cas9 system.^62^ Cells were cultured in DMEM, GlutaMAX (Gibco/Thermo Fisher Scientific), 10% FBS (HyClone GE Healthcare), and 1% penicillin/streptomycin antibiotics (Gibco/Thermo Fisher Scientific) under standard conditions at 37°C with 5% CO_2_. All tumor cell lines were expanded after trypsinization and tested to be mycoplasma-free.

Primary human oral fibroblasts and keratinocytes were obtained from Tebubio (CellResearch Corporation, Singapore). Keratinocytes (hOMK 100) were cultured in DMEM medium supplemented with 10% HyClone and 1% penicillin-streptomycin (P/S) solution under normoxic conditions (37°C, 5% CO_2_). Fibroblasts were maintained in EpiLife basal medium (M-EPI-500-CA + EDGS, Gibco) supplemented with EDGS (S0125, Gibco) and 1% P/S solution, under the same culture conditions. Cells were subcultured at a 1:3 ratio upon reaching 70%–80% confluence after trypsinization.

CAFs derived from a non-FA patient with a tongue squamous cell carcinoma were cultured in DMEM, GlutaMAX (Gibco/Thermo Fisher Scientific), 10% FBS (HyClone GE Healthcare), and 1% penicillin/streptomycin antibiotics (Gibco/Thermo Fisher Scientific) under standard conditions at 37°C with 5% CO_2_. Cells were expanded after trypsinization. To conduct the cytotoxicity tests, HNSCCs, primary cells, and CAFs were transduced with a GFP-luciferase LV, and GFP^+^ cells were then sorted to obtain a pure population of transduced cells.

### Generation of LV-mediated panErbB CAR-T cells

A plasmid containing the T1E-BBz-CAR construct was designed in our laboratory and generated by GenScript (see results and Figure 2A). A plasmid containing the EGFP reporter gene was kindly provided by Prof. L. Naldini and used as a control LV. Plasmids were transformed in *E. coli* strains (Invitrogen) for amplification. Plasmid DNA was purified using Endofree Plasmid Maxi Kit (QIAGEN) following manufacturer’s protocol. In all cases, constructs were verified by enzyme restriction analysis (New England Biolabs). The panErbB CAR construct and the EGFP cDNAs were placed under the control of the eukaryotic translation elongation factor 1α and the phosphoglycerate promoter, respectively. The panErbB CAR and EGFP LVs were generated by a third-generation packaging system using 293T cells that were transiently transfected with calcium chloride with the transfer plasmid pMD.Lg/pPRE (PlasmidFactory), pRSV.REV packaging plasmids, and pMD2.VGVg envelope (PlasmidFactory) plasmid in tissue culture plates at 50%–70% confluence.

LV titers were determined by transduction of 293T cells with serial dilutions of viral supernatants. 7.5 × 10^4^ cells were seeded in 24-well plates 24 h before LV transduction. The same day of titration, cell numbers presented in each well were determined, and serial dilutions of LV supernatants were prepared in IMDM medium for 293T transduction (from 10^−2^ to 10^−6^). Transduced cells were maintained for 14 days. Cells were then collected, and titration was performed by flow cytometry in LSR Fortessa cell analyzer (BD) using FlowJo Software v.10.7.1 (FlowJo, LLC).^69^

For the generation and expansion of panErbB CAR-T cells, fresh or thawed PBMNCs were incubated at a concentration of 10^6^ cells/mL for 4 days with anti-CD3- and anti-CD28-coated nanomatrix (TransAct human, Miltenyi Biotec) to activate T cells and then transduced with panErbB or EGFP LVs at different MOIs in six-well plates (Corning Incorporated). T cells were then transferred to G-Rex24 well plates (Wilsonwolf) and expanded for 10 days in TexMACS medium (Miltenyi Biotec) supplemented with 3% AB human serum and albumin (Sigma-Aldrich), 1% P/S solution (Gibco/Thermo Fisher Scientific), 625 IU/mL IL-7, and 87.5 IU/mL IL-15 (Miltenyi Biotec). Medium was replaced every 3–4 days. Cells were cultured at 37°C under hypoxic conditions (5% O_2_) to preserve the viability of FA cells.^70^ To mimic clinical use, CAR-T cell products were then frozen and maintained in cryopreservation until use. Prior to the *in vitro* or *in vivo* studies with these cells, samples were thawed and used immediately, as would be performed in clinical uses.

### Vector copy-number determinations

Genomic DNA was extracted using DNA purification Kit NucleoSpin Tissue (Machery-Nagel) following manufacturer’s instructions. Quantitative PCR analyses were performed to evaluate the VCN/cell, based on the simultaneous amplification of Ψ (psi) viral region and human ALBUMIN gene, as endogenous genome reference, using ABsolute qPCR Mix, low ROX (Thermo Fisher Scientific #AB1318). Specific primers were used for the psi sequence (psi forward [5′ CAGGACTCGGCTTGCTGAAG 3′] and psi reverse [5′ TCCCCCGCTTAATACTGACG 3′]) and detected with the TaqMan probe (5′ CGCACGGCAAGAGGCGAGG 3′). To normalize to endogenous ALBUMIN, specific primers for ALBUMIN were used (Alb forward [5′ GCTGTCATCTCTTGTGGGCTG 3′] and Alb reverse [5′ ACTCATGGGAGCTGCTGGTTC 3′]), together with the TaqMan probe (5′ CCTGTCATGCCCACACAAATCTCTCC 3′). qPCR was conducted in an Applied Biosystems 7500 Fast Real-Time PCR system (Thermo Fisher Scientific). A synthetic double-stranded DNA fragment (gBlock) containing Ψ and album sequences was used as standard curve.

### Monoclonal antibodies and flow cytometry analyses

To analyze the expression of ErbB receptors in the membrane of HNSCC cell lines, the following antibodies were used: EGFR APC (BioLegend), ErbB2 (HER-2) biotin (Invitrogen), ErbB3 biotin (Invitrogen), and ErbB4 biotin (Invitrogen). As a secondary antibody for staining ErbB2, ErbB3, and ErbB4, a donkey anti-rabbit IgG A647 was used.

The following antibodies were used to analyze T cell differentiation subpopulations and activation markers: CD4 FITC (Beckman Coulter), CCR7 PE (BD Pharmingen), CD45RA PerCP-Cy5.5 (BioLegend), CD3 PE-Cy7 (BioLegend), CXCR3 APC (BioLegend), CD8 BV510 (BioLegend), CD3 FITC (Dako), CD69 PE (BioLegend), ICOS PerCP-Cy5.5 (BioLegend), HLA-DR PE-Cy7 (BioLegend), and CD137 APC (BioLegend). Isotype controls in this analysis were IgG1 PE (BioLegend), Hamster IgG PerCP-Cy5.5 (BioLegend), IgG2a PE-Cy7 (BioLegend), and IgG1 APC (BioLegend). For exhaustion markers, the antibodies used were CD3 FITC (Dako), LAG3 PE (BD Pharmingen), PD1 PerCP-Cy5.5 (BioLegend), TIGIT PE-Cy7 (BioLegend), CTLA4 APC (BioLegend), TIM3 APC-Cy7 (BioLegend), and CD8 BV510 (BioLegend). Isotype controls for these antibodies were IgG1 PE (BioLegend), IgG1 PerCP-Cy5.5 (BioLegend), IgG2a PE-Cy7 (BioLegend), IgG1 APC (BioLegend), IgG1 APC-Cy7 (BioLegend), and CD8 BV510 (BioLegend).

Percentages of transduction were analyzed using CD3 PE (Dako), hEGFb (Human EGF Biotinylated Affinity Purified PAb, Bio-Techne, R&D Systems, Inc.) as primary antibody, and BV-711 streptavidin (BD Pharmingen) as a secondary marker; and viability was studied with PBS with 0.5% BSA and 0.005% sodium azide at 1 μg/mL of 4′,6-diamidino-2-phenylindole (DAPI, Thermo Fisher Scientific). Flow cytometry analyses were conducted in an LSR Fortessa cell analyzer (BD/Becton Dickinson and Company, New Jersey, USA). Analyses were performed with FlowJo Software v.10.7.1 (FlowJo, LLC). A minimum number of 5,000 viable cells were acquired in the LSR Fortessa.

### *In vitro* cytotoxicity tests

To test the cytotoxic potential of CAR-T cells *in vitro*, 25,000 HNSCC cells expressing GFP/luciferase were cultured in 96-well plates (Poly-D-lysine Cellware, Corning, BioCoat). After 5 hours, CAR-T cells were added in triplicates at different E:T ratios (final volume of 200 μL), including HNSCCs alone as a control of 100% of viability. The luminescence in each well was determined 24, 48, 72, and 96 h after co-culture. XenoLight D-Luciferin-K+ salt bioluminescent substrate (PerkinElmer) was then added at a concentration of 15 μg/mL. After 10 min incubation at 37°C, the luminescence signal was read in the Tecan Infinite 200 Pro M Plex microplate reader. The cytotoxicity was then calculated as % cytotoxicity = 100 − % live cells (100 × [(luminescence well)/(average luminescence of tumor cells alone)]).

### Cytokine quantification

At the end of the CAR-T cell manufacturing, 1 × 10^6^ cells were transferred to a well containing 1 mL of fresh medium, and cytokine secretion was analyzed after 24 h. Cytokine levels were also measured in co-cultures of CAR-T cells with the VU-1131 cell line at an E:T ratio of 1.25:1 in a total volume of 1 mL, with samples collected at 24 and 72 h. Three human-secreted proteins (IL-2, IFN-γ, and TNF-α) were analyzed in triplicates by flow cytometry using RayPlex Human Cytotoxic T cell Array from RayBiotech, following manufacturer’s instructions.

### Animal models

*In vivo* studies were conducted using 8- to 12-week-old female non-obese diabetic Cg-Prkdcscid Il2rgtm1Wjl/SzJ (NGS) mice housed in micro-isolator boxes with controlled temperature, humidity, and light cycles at the CIEMAT animal facility under specific pathogen-free condition. Mice were provided with standard autoclavable rodent chow. All experimental procedures and protocols were reviewed and approved by the Institutional Animal Care and Use Ethics Committee of the CIEMAT (PROEX 206.1-23), and the “Guide for the Care and Use of Laboratory Animals” was followed carefully. Ethical limits for the experiments were based on weight loss, posture (hunching), mobility, tumor size, and the presence of extensive tumoral necrosis. Mice were used without prior irradiation and were subcutaneously inoculated in both flanks to establish tumor xenografts with 5 × 10^6^ VU-1131-GFP-luc HNSCCs. Once tumors reached bioluminescence signals of 3 × 10^9^ to 5 × 10^9^ photons/s, mice were divided into three groups (PBS, EGFP^+^ T lymphocytes, and panErbB CAR-T cells), with each group containing comparable tumor burden measurements. A single dose of 15 × 10^6^ FA or HD panErbB CAR-T cell manufacturing products or EGFP^+^ T lymphocytes was intratumorally injected into each tumor in a volume of 100 μL. Tumor burden was then quantified at designated time points by BLI, performed 10 min after intraperitoneal injection of luciferin (52 mg/kg). Imaging was conducted using the IVIS Lumina XRMS Series III (PerkinElmer) platform, and the data were analyzed with the Living Image software, reported as average flux [photons per second/area (mm^2^)]. Mice were anesthetized with isoflurane gas during the imaging procedure. Tumor growth was monitored twice a week until tumor burden exceeded 3 × 10^10^ to 8 × 10^10^ p/s.

At the end of the experiment, mice were euthanized, and intracardiac blood was collected for flow cytometry analysis of CAR-T cells. Tumors were excised and processed to isolate human tumor-infiltrating cells. Tumor tissue was treated enzymatically with 2 mg/mL collagenase (COLLA-RO, Merck) and mechanically disaggregated using scalpels. The resulting tumor cell suspension was filtered to remove debris. Nuclear cells were analyzed by flow cytometry to determine the presence of hCD3^+^ and CAR-T cells.

### Immunohistochemistry in tumor xenografts

Mouse tissues were fixed in 4% buffered formalin and embedded in paraffin. Sections (5 μm) were stained with H&E or processed for immunohistochemistry. Primary antibodies used were anti-EGFR monoclonal antibody (no. 4267, Cell Signaling, 1:20) and anti-Erbb2 monoclonal antibody (no. UMAB36, Origene, 1:20). Secondary antibodies for immunohistochemistry were used as follows: biotin anti-rabbit (no. 711- 065-152, Jackson ImmunoResearch, 1:1,000) and biotin anti-mouse (no. 715-065-151, Jackson ImmunoResearch, 1:1,000). Immunoreactivity was revealed using the ABC-peroxidase system and the DAB substrate kit (Vector Laboratories, Burlingame, CA, USA), and the sections were counterstained with hematoxylin. Control experiments without the primary antibody gave no signal. Images were captured using Leica optical microscope (DM2000 LED) and LAS X software (v.3.7.4.23463, Leica Microsystems, Wetzlar, Germany).

### Statistics

Normality of data distribution was analyzed by the Shapiro-Wilk’s test. To determine statistically significant differences between independent continuous variables, a 2-tailed paired or unpaired Student’s *t* test with Welch’s correction was applied when an equal SD was not assumed for normal distribution, and a 2-tailed, nonparametric Mann-Whitney U test was used for data with a non-normal distribution. For multiple comparisons, the Kruskal-Wallis test was followed by Dunn’s multiple-comparison test. Spearman’s rank correlation coefficient was used to analyze associations between 2 continuous variables. Statistical analyses were performed with GraphPad Prism, version 9.5.1 (GraphPad Software), and *p* values lower than 0.05 were considered statistically significant.

## Data availability

The datasets generated and/or analyzed during this study are available from the corresponding author upon reasonable request.

## Acknowledgments

This work was supported by grants from “Red Española de Terapias Avanzadas RICORS/TERAV (RD21/0017/0027)”, “RICORS/TERAVplus (RD24/0014/0023)” from 10.13039/501100004587Instituto de Salud Carlos III (ISCIII), and by grants from the Spanish Government co-financed by Fondo Europeo de Desarrollo Regional (FEDER) PI21/00208 and 10.13039/501100014139CIBERONC no. CB16/12/00228 from the Instituto de Salud Carlos III (ISCIII); and a grant from Fundación Anemia de Fanconi to R.G.-E. The work has been conducted within the framework of an Agreement between 10.13039/501100004587Instituto de Salud Carlos III and 10.13039/501100009613CIEMAT for the creation of a Mixed Unit on Advanced Therapies (Resolution 27/1/2025).

The authors thank A. de la Cal, J. Reig, and A. Baizán for coordinating the delivery of PB samples from patients with FA. The authors thank M. Lopez for her help with the statistical analyses. The authors also thank the CIEMAT Health Department for providing PB samples from healthy donors. The authors are also indebted to the patients with FA, their families, and clinicians from the Fundación Anemia de Fanconi.

## Author contributions

A.L. analyzed data and wrote the manuscript. A.L., D.C., and P.V. performed *in vitro* and *in vivo* experiments. B.D. generated the panErbB LV. P.R. and C.L. provided conceptual guidance. R.S. and O.A. helped and supported with the flow cytometry studies. B.M.-A. and E.E. provided the GFP/luciferase LV and helped with *in vitro* and *in vivo* cytotoxic experiments. R.G.-E., R.E., S.D.M., and A.P. provided FA-defective HNSCC cell lines, helped with the mouse models, shared their knowledge of FA-HNSCC, and helped with the immunohistochemistry analyses of the tumors. J.M. provided the T1E28z gamma-retroviral vector and provided guidance. J.A.B. identified unmet medical needs, provided conceptual guidance, and wrote the manuscript. J.A.C. supervised the project, including the design of the experiments and the data analysis, and wrote the manuscript. All authors revised the manuscript.

## Declaration of interests

P.R. and J.A.B. have received honoraria as consultants and hold stock options and royalties for licenses to Rocket Pharmaceuticals for the gene therapy of patients with FA. J.M. is a founding scientist, shareholder, and chief scientific officer of the CAR-T cell company, Leucid Bio.

## Declaration of generative AI and AI-assisted technologies in the writing process

During the preparation of this work, the author(s) used ChatGPT to review certain sections for spelling and grammar errors. Following the use of this tool, the author(s) carefully reviewed and edited the content as necessary, taking full responsibility for the final version of the publication.

## Supplemental information

Document S1. Figures S1–S6

Document S2. Article plus supplemental information
