# Supplementary material for: Lentiviral-mediated panErbB CAR-T cell therapy against head and neck squamous cell carcinomas for patients with Fanconi anemia
Source: Mol Ther Oncol. 2025 Sep 22;33(4):201060. doi: 10.1016/j.omton.2025.201060 (PMC12547774; doi:10.1016/j.omton.2025.201060)
Supplement: Document S1. Figures S1–S6 [file mmc1.pdf]

OMTON, Volume *33*

Supplemental information

Lentiviral-mediated panErbB CAR-T cell therapy against head and neck squamous cell carcinomas for patients with Fanconi anemia

Andrea López, David Charbonnier, Paula Vela, Begoña Díez, Paula Río, Rebeca Sánchez, Omaira Alberquilla, Beatriz Martín-Antonio, Jordi Minguillón, Esperanza Esquinas, Ramón García-Escudero, Ricardo Errazquin, Sonia Del Marro, Ania Pascual, Corina Lorz, Ángeles Juarranz, Andrea Barahona, Judith Balmaña, John Maher, Juan A. Bueren, and José Antonio Casado

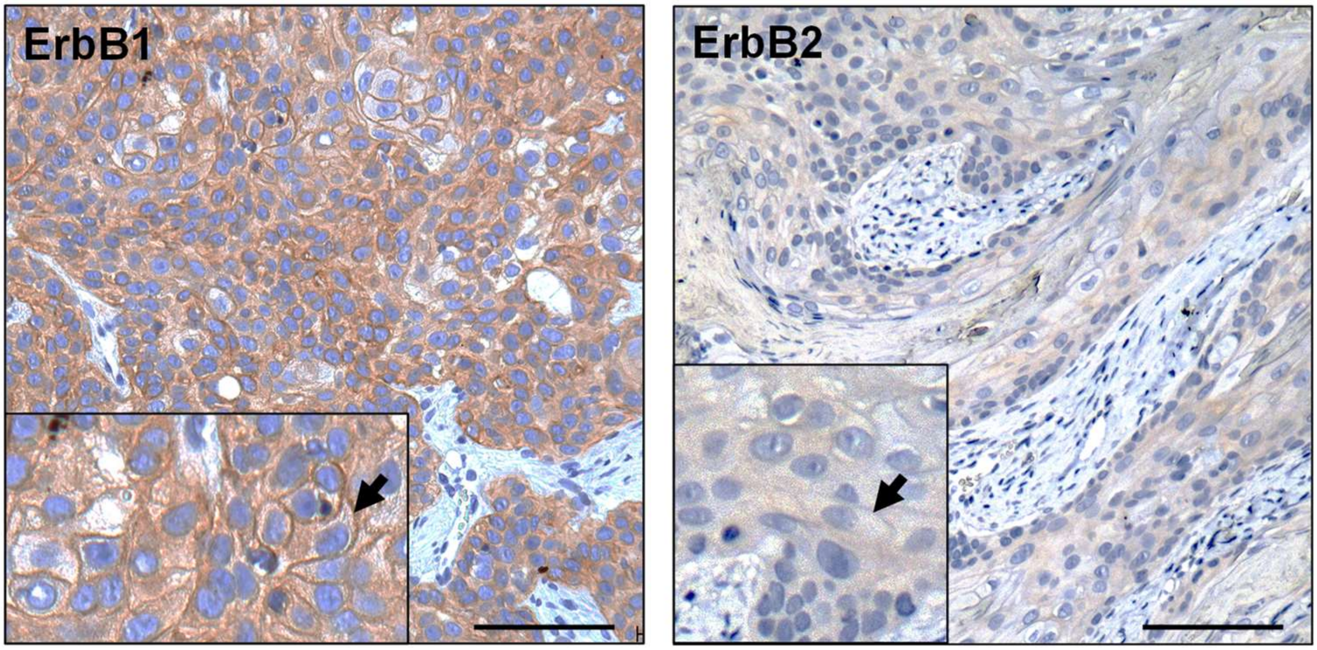

Figure S1. Immunohistochemistry analysis of the expression of ErbB1 (EGFR) and ErbB2 in HNSCC tumors. The expression of ErbB1 and ErbB2 receptors is shown in carcinoma cells from VU-1131 tumors after xenotransplantation into NSG mice. The staining pattern is evidenced in the cytoplasm and the cell membrane of the HNSCC (indicated by arrows in the insets). Scale bars: 100 µm.

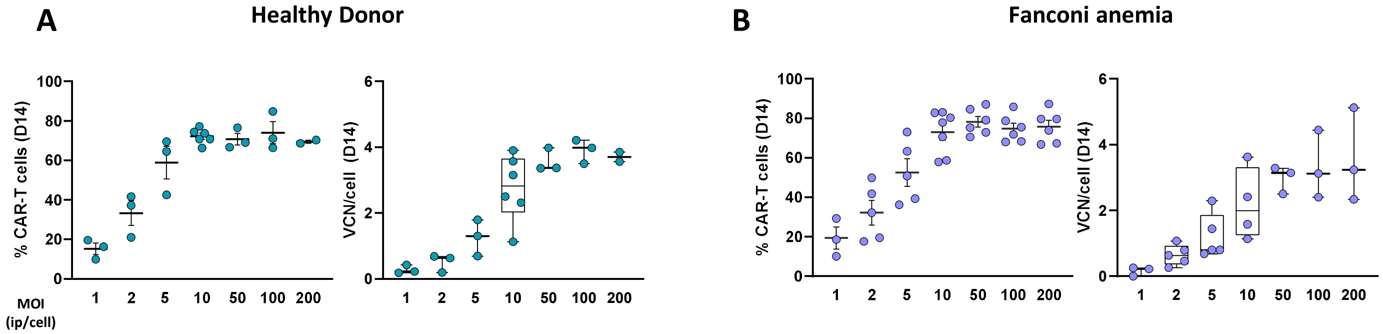

Figure S2. Optimization of the multiplicity of infection to generate CAR-T cells with the pan-ErbB lentiviral vector. The figure shows the mean ± SEM of the transduction efficacy (left panels) and the min to max plot vector copy number per cell (VCN/cell; right panels) corresponding to day 14 of the manufacturing process using different MOIs of the panErbB LV. **A)** Samples from healthy donors: MOI 10 (n=6), for the rest of MOIs (n=3). **B)** Samples from patients with FA. For the % of CAR-T cells: MOI of 1 (*n*=3); MOIs of 2 and 5 (*n*=5); MOI of 10 (*n*=7); MOIs of 50, 100, 200 (*n*=6). For the VCN/Cell: MOI of 1 (*n*=3); MOIs of 2 and 5 (*n*=5); MOI of 10 (*n*=4); MOIs of 50, 100 and 200 (*n*=3).

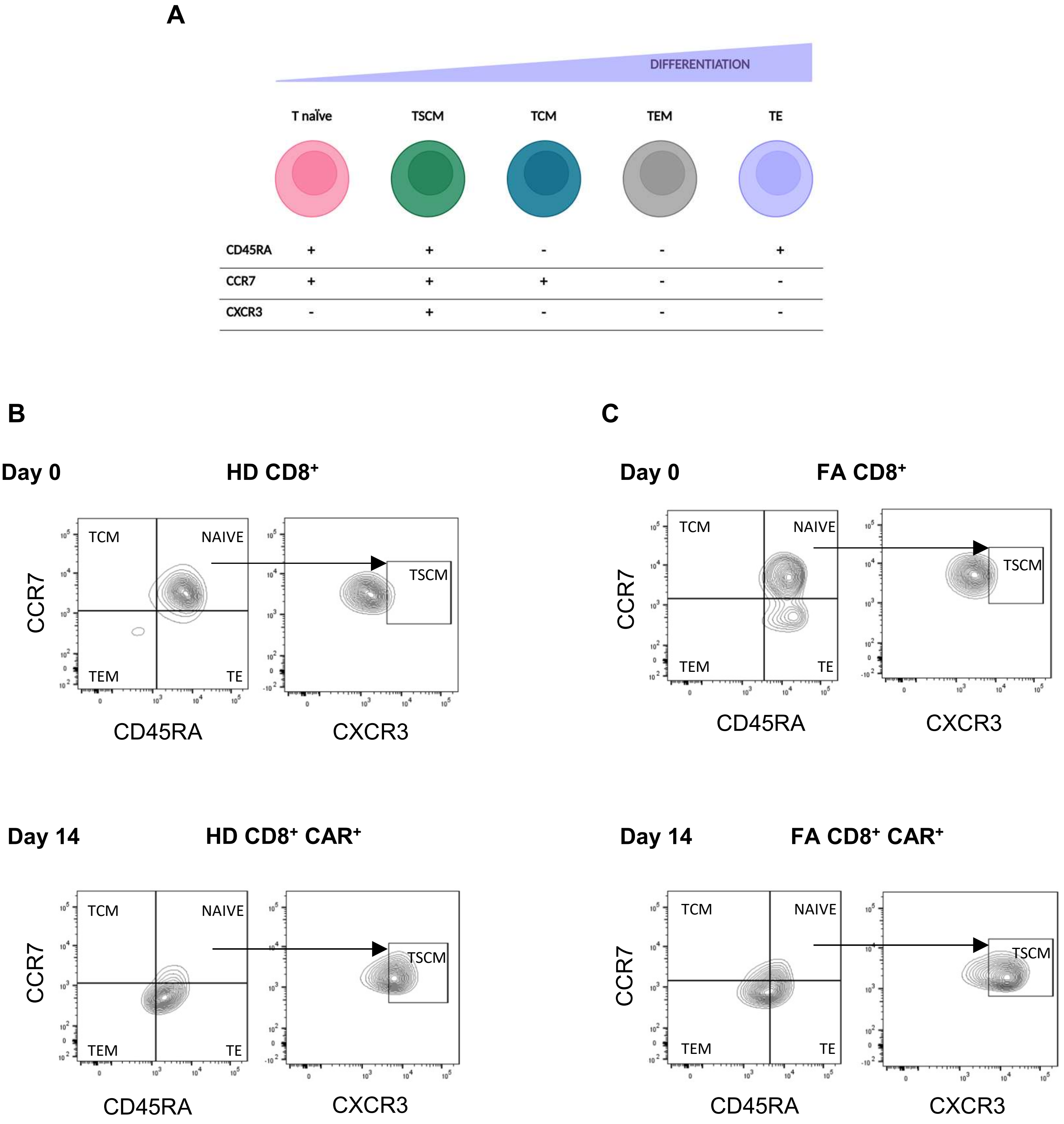

Figure S3. Representative flow cytometry analyses of differentiation markers shown in Figure 3F. A) Schematic representation of T Cell differentiation markers (CCR7, CD45RA and CXCR3). Panels **B)** and **C)** show, respectively flow cytometry analyses corresponding to samples from a healthy donor and an untrasplanted Fanconi anemia patient. In each case, T cell subpopulations were analyzed at day 0 ($CD8^+$ cells) and day 14 ($CD8^+CAR^+$ T cells).

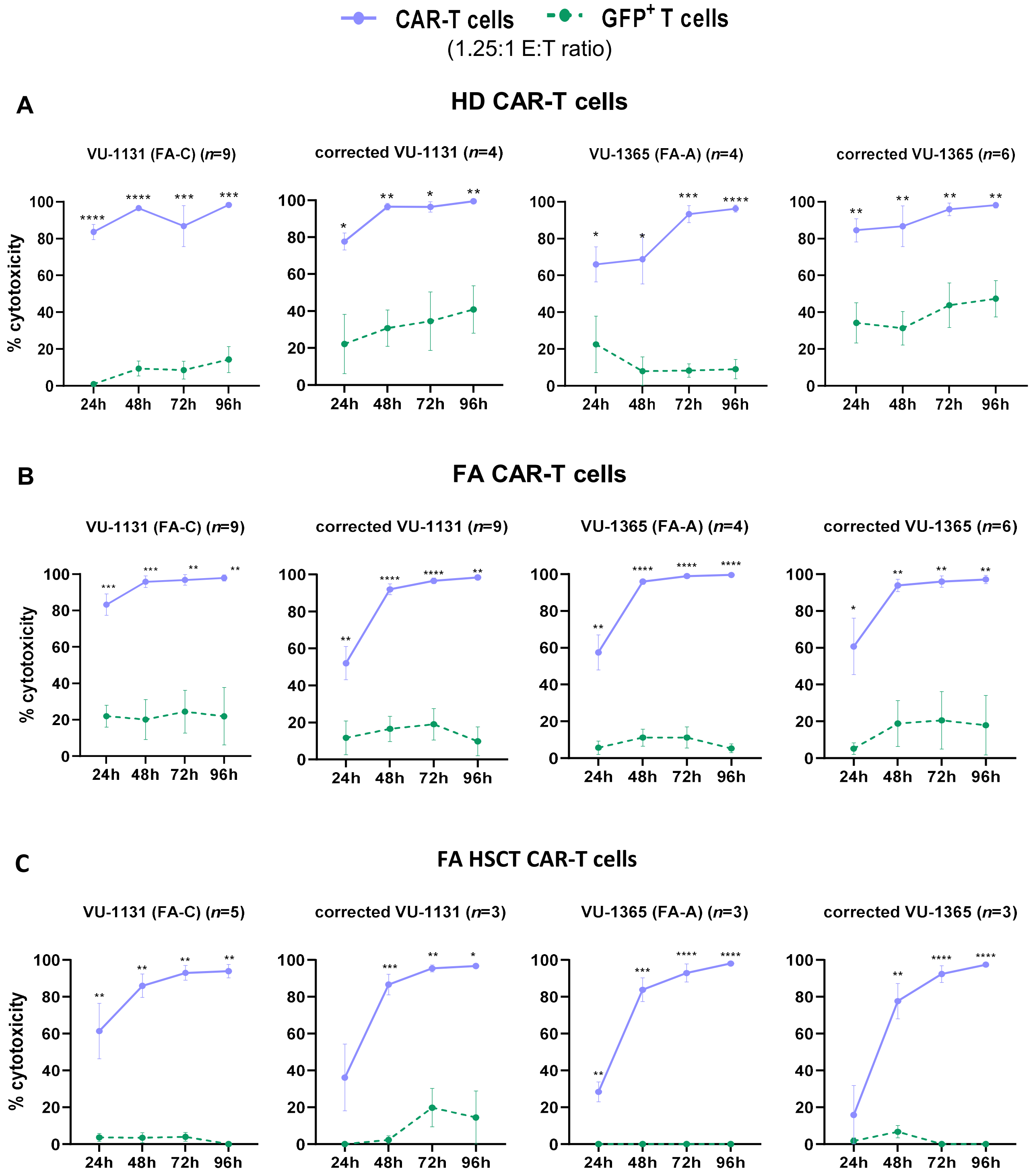

Figure S4. In vitro cytotoxicity of healthy donor and Fanconi anemia panErbB CAR-T and EGFP-transduced T-lymphocytes against HNSCC cell lines. For each sample, CAR T cells and EGFP T-lymphocytes were co-cultured with HNSCCs. Data show the cytotoxicity of CAR-T cells compared to EGFP-transduced T cells against HNSCC lines at a 1.25:1 ratio. Each dot represents the mean ± SEM corresponding to different samples (*n* indicated at the top of each graph). **A)** Cytotoxicity against HNSCC cell lines of HD CAR-T EGFP-transduced T cells co-cultured with VU-1131, VU-1131+FANCC, VU-1365 and VU-1365+FANCA. **B)** Cytotoxicity against HNSCC cell lines of FA CAR-T or EGFP-transduced T cells co-cultured with the same four HNSCC cell lines. Statistical analyses performed following Mann-Whitney or t Student test after Saphiro-Wilk´s test for unpaired comparisons and represented in the legend relative to day 19. **p < 0.01; ***p < 0.001; ****p < 0.0001.

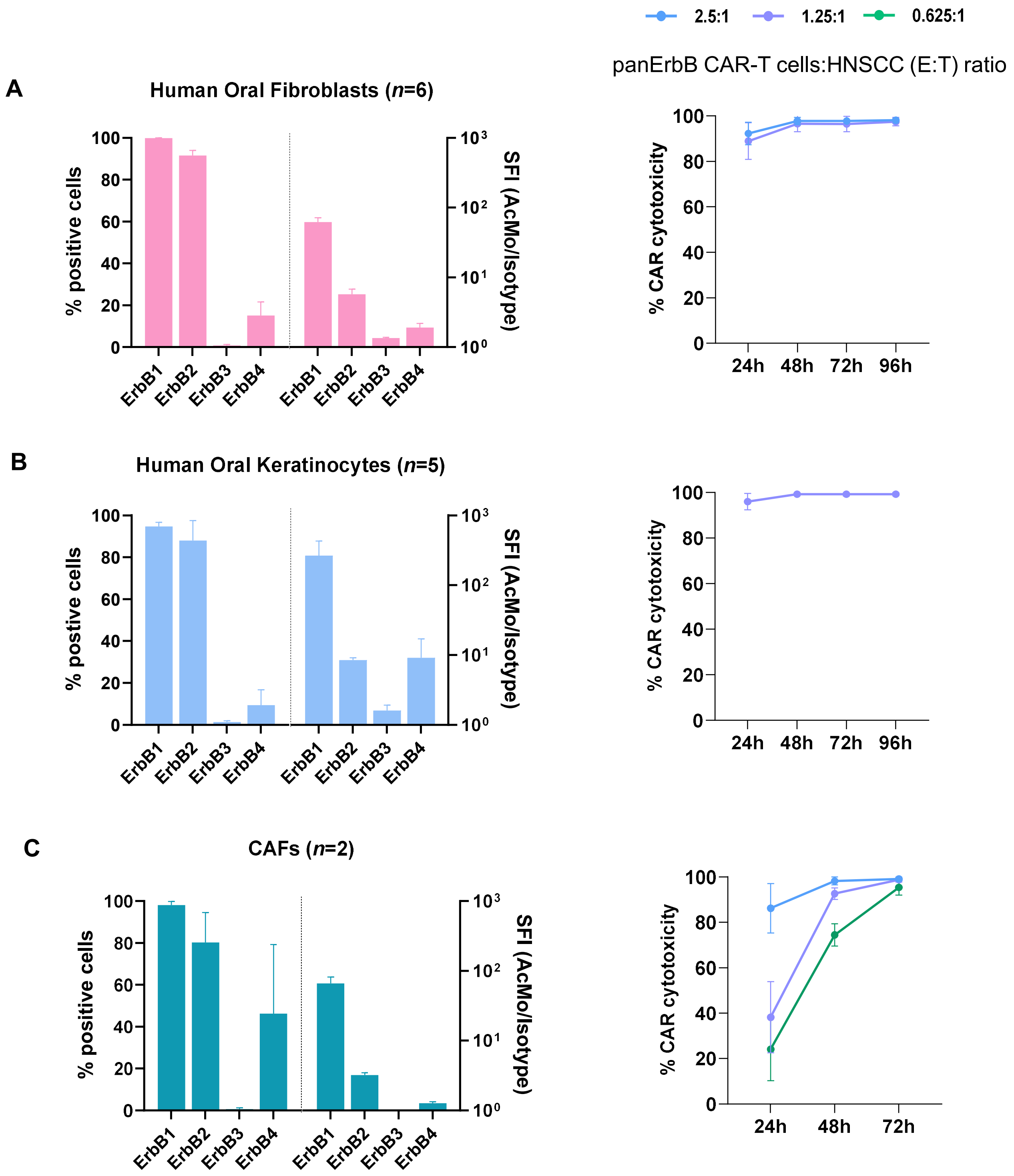

Figure S5. "On-target, off-tumor" *in vitro* activity of panErbB CAR-T cells in primary oral human fibroblasts, keratinocytes and cancer-associated fibroblasts. The left part of the panel represents the analysis of ErbB molecules (left and right axes represent, respectively, the percentage of ErbB positive cells and the specific fluorescence intensity (SFI)). The SFI expression levels of the different ErbB members were the ratio between the mean fluorescence intensity (MFI) after staining with the corresponding monoclonal antibody (MoAb) relative to MFI obtained with the control isotype. Bars show the mean ± SEM; *n* is indicated at the top of each graph. The right part of the panel represents the percentage of cytotoxicity of CAR-T cells against the three different primary cells. Dots represent the mean ± SEM. **A)** Primary human oral fibroblasts. **B)** Primary human oral keratinocytes. **C)** Cancer-associated fibroblasts (CAFs).

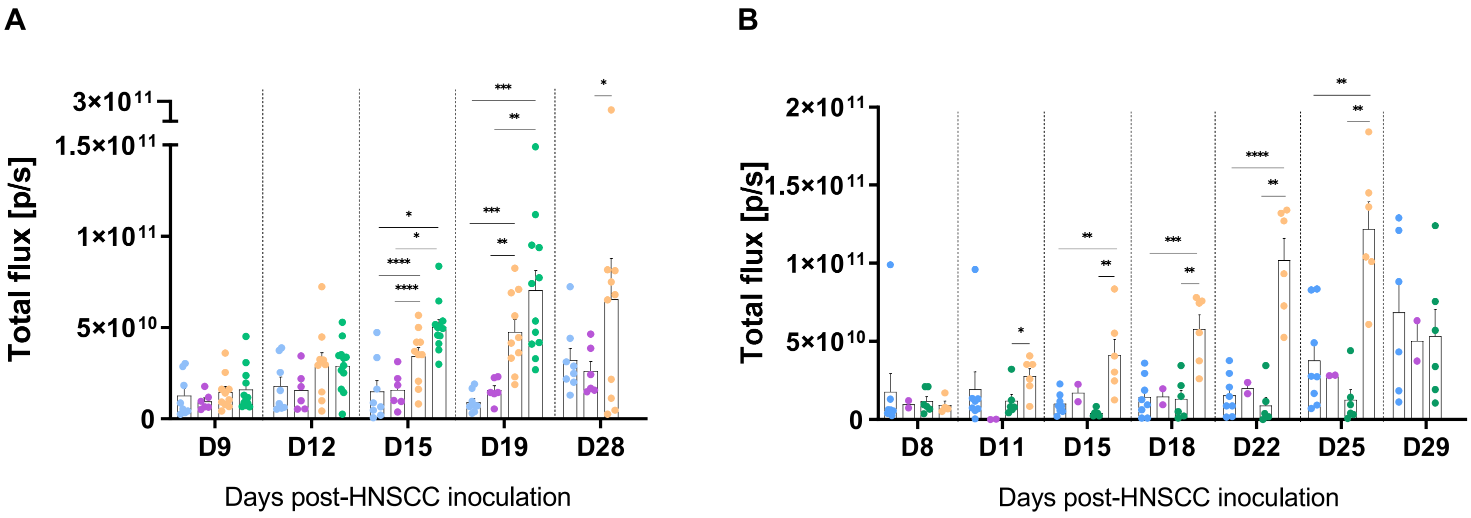

Figure S6. Individual data of *in vivo* luminescence corresponding to each tumor. A) Individual data corresponding to Figure 6B, from the *in vivo* experiment with FA CAR-T panErbB cells. **B)** Individual data corresponding to Figure 7B, from the *in vivo* experiment with both HD and FA CAR-T panErbB cells. Statistical analyses performed following Mann-Whitney or t Student test after Saphiro-Wilk´s test for unpaired comparisons. **p < 0.01; ***p < 0.001; ****p < 0.0001.
